# Safety and Accuracy of Core Needle Biopsy for Soft Tissue Masses in an Ambulatory Setting

**DOI:** 10.1155/2018/1657864

**Published:** 2018-06-12

**Authors:** J. Brock Walker, Erin Stockwell, Kellen Worhacz, Paul Kang, Amalia Decomas

**Affiliations:** ^1^University of Arizona College of Medicine, Phoenix, AZ, USA; ^2^The CORE Institute, Phoenix, AZ, USA

## Abstract

**Background:**

Percutaneous needle biopsy has been found to be a safe and accurate method for the initial investigation of soft tissue masses. The notion exists that needle biopsies should be performed in specialized sarcoma centers, which can place a financial burden on patients without a sarcoma center near their place of residence. There is no consensus in the current literature regarding the diagnostic accuracy and clinical utility of clinic-based percutaneous core needle biopsy performed by community orthopedic surgeons with fellowship training in musculoskeletal oncology.

**Questions/Purposes:**

Our primary goal was to determine if office-based core needle biopsy of soft tissue masses could safely yield accurate diagnoses when performed by a community orthopedic surgeon with fellowship training in musculoskeletal oncology.

**Patients and Methods:**

We retrospectively reviewed the charts of 105 patients who underwent percutaneous core needle biopsy of soft tissue masses in a community clinic. All procedures were performed by one fellowship-trained musculoskeletal oncologist. Accuracy of the initial clinic-based needle biopsy was determined through comparison to the results of pathological analysis of the surgically excised masses. Final data analysis included 69 patients who underwent both clinic-based biopsy and subsequent surgical excision of their masses.

**Results:**

We found clinic-based biopsies to be 87.0% accurate for exact diagnosis and 94.2% accurate in determining whether the mass was benign or malignant (*p* < 0.0001). Minor complications related to the clinic-based biopsy occurred in 5.80% of cases, with no documentation of major complications.

**Conclusions:**

Our results provide evidence that office-based percutaneous biopsy can be administered safely and yield accurate, clinically useful results when performed by a fellowship-trained musculoskeletal oncologist.

## 1. Introduction

### 1.1. Background

Soft tissue sarcomas are exceedingly rare tumors with a mesenchymal origin [1]. They most commonly occur in the soft tissues of extremities and present in pediatric patients more often than adults [2]. Prompt, accurate diagnosis of soft tissue masses can be critical in initiating treatment of these tumors, which can carry significant morbidity and mortality. Conventional diagnosis of soft tissue masses through open incisional biopsy has been shown to give accurate diagnoses in 91% to 96% of cases [3–8]. However, this technique has demonstrated increased rates of complications [4, 6, 9, 10] over less invasive biopsy techniques such as percutaneous core needle biopsy (CNB) or fine-needle aspiration (FNA) [3, 7, 11–13]. For this reason, percutaneous CNB has become increasingly common for initial biopsy of soft tissue masses, exhibiting accuracy rates of 80% to 98% [7, 11, 13–22].

### 1.2. Rationale

It has been suggested by multiple authors that percutaneous soft tissue biopsies should be performed at sarcoma referral centers under the care of experienced musculoskeletal oncologists, citing decreased accuracy and potential alterations in the clinical course when biopsies are performed in the community [7, 13, 15, 23–25]. However, many of these studies failed to account for fellowship-trained musculoskeletal oncologists that practice in community centers. Currently, there is no consensus in the orthopedic literature on percutaneous CNB of soft tissue masses performed in community clinics by experienced musculoskeletal oncologists. The goal of our study is to retrospectively examine the diagnostic accuracy of office-based percutaneous core needle biopsy (CNB) when performed by a community orthopedic surgeon with fellowship training in musculoskeletal oncology. Secondarily, we aim to determine if correct treatment would have been guided if only the clinic-based biopsy were performed.

## 2. Methods

### 2.1. Study Design and Setting

This retrospective chart review was performed under Institutional Review Board (IRB) approval with waiver of informed consent. The initial list of patients was generated using ICD and CPT codes related to percutaneous needle biopsy. All data were collected from the electronic medical records (EMRs) of one community orthopedic clinic and one large academic medical center.

### 2.2. Participants

**Figure 1:**
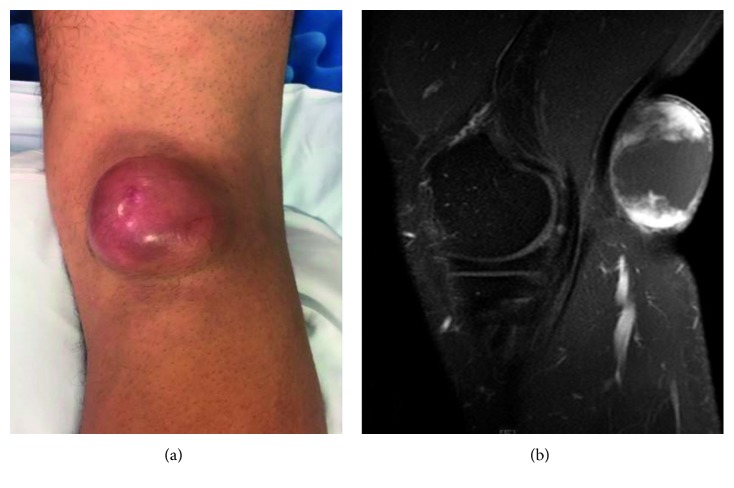
The image shows a 40-year-old male with a painless, superficial popliteal mass seen on (a) physical examination and (b) magnetic resonance imaging. In-office biopsy results showed intermediate-grade soft tissue sarcoma of indeterminate type. Final surgical pathology results showed grade 2 fibrosarcoma.

We reviewed the charts of all patients who underwent ambulatory percutaneous needle biopsy of a soft tissue mass (Figure 1) between April 2011 and February 2017 under the care of a single board-certified orthopedic surgeon with fellowship training in musculoskeletal oncology (*n*=105). Patients who did not ultimately undergo surgical excision of their lesions were excluded from further data collection and analysis.

### 2.3. Procedure

All procedures of office-based percutaneous needle biopsy and final surgical excision of soft tissue lesions were performed by a single board-certified and fellowship-trained musculoskeletal oncologist. Informed consent was obtained following a thorough discussion of risks, benefits, and expectations prior to the completion of any procedures. Needle biopsies were performed with Tru-Cut^©^ needles (Allegiance, Illinois, USA), taking multiple cores to maximize the amount of tissue biopsied, thus increasing the diagnostic ability [26]. Image guidance was not used in the biopsy procedure. Final surgical excisions were performed in the operating room under general anesthesia. All histopathological sections were examined by experienced, board-certified pathologists. Pathology results were reported in accordance with the 2013 World Health Organization bone and soft tissue tumor guidelines [27].

### 2.4. Variables, Outcome Measures, Data Sources, and Bias

Data collection from the final study population (*n*=69) included the following:

1. Patient demographic information (Table 1)
2. Lesion location
3. Lesion diameter
4. Lesion depth relative to fascia (superficial or deep)
5. Result of percutaneous biopsy (benign or malignant, exact diagnosis)
6. Result of final surgical pathological analysis (benign or malignant, exact diagnosis)
7. Documentation of biopsy-related complications
8. Documentation of the propriety of patients' ultimate treatment

The primary outcome examined was the diagnostic accuracy of the office-based percutaneous needle biopsy. This was obtained through examination of the level of agreement between the results of the initial office-based biopsy and pathological analysis of the surgically excised soft tissue lesion. Agreement between the two biopsies was determined based on both the status of the lesion (benign versus malignant) and the exact diagnosis. All pathology reports were reviewed by a third-party board-certified pathologist in order to determine the accuracy of ambulatory percutaneous biopsy when compared to the final pathological analysis of the surgically excised masses. In order to assess safety, we included documentation of any complications determined to be related to the ambulatory biopsy. Clinical utility of the clinic-based biopsy as a diagnostic test was assessed through determination of whether appropriate clinical treatment for each patient's final diagnosis would have been initiated with office-based CNB as the only diagnostic tool.

### 2.5. Statistical Analysis

All statistical analysis was performed using Stata (StataCorp, Texas, USA). Accuracy was determined through the percent agreement between the exact diagnoses given by each biopsy method. Spearman's rank correlation coefficient (Spearman's rho) was used to quantify the level of agreement between the two biopsy techniques (benign versus malignant) and determine statistical significance of the primary outcome (*α* = 0.05). This statistical test is used to quantify the correlation between two nonparametric variables [28]. Sensitivity, specificity, positive predictive value (PPV), and negative predictive value (NPV) were calculated for the office-based biopsy. Results were compared against the final pathological examination of the surgically excised lesions (used as gold standard) to determine these measures. For example, lesions determined to be benign by both biopsy methods were designated as true negatives (TNs). If a lesion was determined to be benign through office-based percutaneous needle biopsy but malignant when examined after surgical excision, this result was designated a false negative (FN).

## 3. Results

### 3.1. Safety and Accuracy (including Information on Accuracy and Complications)

Demographic information of the 69 patients included in our final data analysis is described in Table 1. The exact pathological diagnosis accuracy of our clinic-based CNB was determined to be 87.0% based on the percent agreement between the CNB and final surgical pathology. The correct nature of the mass (benign versus malignant) was identified in 94.2% of patients. This was determined to be significant through Spearman's correlation coefficient (*p* < 0.0001). Accuracy results are further stratified by gender, lesion location, lesion size, and depth of lesion in Table 1. Minor complications related to the CNB procedure occurred in 3 patients (4.3%) including hematoma in 2 patients and bleeding in 1 patient requiring placement of 1 suture in the clinic. No major complications occurred as a result of the CNB.

In our study, 4 patients had malignant lesions that were reported benign by CNB, and thus these biopsies were counted as incorrect for both exact diagnosis and determining benign versus malignant. These patients were as follows:

- A 45-year-old female with a left distal lateral arm mass. Initial CNB was read as “adipose and fibrous tissue with focal myxoid change.” Due to a high clinical suspicion and the fact that this mass could be excised with adequate margins without increased morbidity, a wide resection was performed. Final pathology reported the mass as a grade 1 myxoid liposarcoma.
- A 60-year-old male with a mass about the left proximal posterior calf, enveloping the distal popliteal neurovascular bundle. Initial biopsy at the margins of the lesion reported “spindle cell neoplasm.” Due to high clinical suspicion for malignancy, the nonspecific nature of the biopsy report, and involvement of critical neurovascular structures, the patient was given the option of open biopsy for further diagnosis. However, the patient declined this and opted for above knee amputation. The final pathology report diagnosed a “malignant solitary fibrous tumor.”
- A 55-year-old female with a mass about the right shoulder. Initial biopsy reported a “low-grade spindle cell lesion.” Due to high clinical suspicion and the nonspecific results of the biopsy, an open biopsy was performed at the time of planned excision. This frozen section was read as high-grade sarcoma, and thus a wide excision was performed. Final pathology was reported as a high-grade pleomorphic sarcoma.
- A 56-year-old male with a mass to the right anterior thigh. Initial biopsy reported skeletal muscle and mature adipose tissue. Following marginal excision, the final pathology report indicated the mass was a well-differentiated liposarcoma.

Additional 5 biopsies did not report the exact diagnosis, but were correct with regards to benign versus malignant. These patients were as follows:

- A 46-year-old female with a left shoulder mass. Initial CNB was read as “fragments of skeletal muscle and adipose tissue”; however, final pathology after marginal resection reported the mass as a desmoplastic fibroblastoma.
- A 45-year-old male with a mass about the left arm. Initial CNB reported “benign skeletal muscle.” After marginal resection, final pathology diagnosed a benign vascular malformation.
- A 55-year-old male with a mass to the posterior elbow. Initial biopsy reported benign skeletal and adipose tissue. This mass was marginally excised, and the final pathology report diagnosed a “benign vascular malformation with thrombosis and papillary endothelial hyperplasia.”
- A 50-year-old male with a mass to the right lateral thigh. Initial biopsy reported a benign lipomatous lesion. Due to clinical suspicion and the ease of wide resection without increased morbidity, wide resection was performed. The final pathology report diagnosed the mass as a “hibernoma with myxoid features.”
- A 84-year-old male with a mass to the right posterior thigh. Initial biopsy reported a “bland fibromyxoid spindle cell neoplasm.” Due to high clinical suspicion and the ease of wide resection without increased morbidity, wide resection was performed. The final pathology report diagnosed the mass as a perineuroma.

Upon retrospective review of all of these cases, the use of office-based core needle biopsy, in combination with clinical suspicion and the optional use of an open biopsy, leads to the correct treatment in 100% of cases.

### 3.2. Clinical Utility (including Information on Appropriate Treatment and Sensitivity)

We also assessed the clinical utility of our office-based biopsy in terms of a diagnostic test for malignancy. Overall sensitivity, specificity, PPV, and NPV were found to be 87.1%, 100.0%, 100.0%, and 90.5%, respectively. Using the results of the pathological analysis of the final surgical excision, we determined that all patients received correct treatment following the use of the office-based CNB.

## 4. Discussion

### 4.1. Background and Rationale

Percutaneous needle biopsy has been shown by numerous studies to be safe and effective for diagnosis of soft tissue tumors when compared to open biopsy [3, 7, 11, 13–18, 20, 22, 29, 30]. However, the previous literature has recommended against performing these diagnostic procedures in the community setting because of the possibility of complications or inaccuracy resulting in inappropriate subsequent treatment [7, 13, 15, 23]. Our study aimed to challenge this notion and provide evidence that accurate and clinically useful percutaneous CNB could be performed outside a sarcoma center by community orthopedic surgeons with fellowship training in musculoskeletal oncology.

Currently, there is no consensus in the literature regarding the accuracy and clinical utility of office-based CNB of soft tissue masses. Skrzynski et al. examined the diagnostic accuracy and financial burden of musculoskeletal tumor biopsy, comparing clinic-based percutaneous needle techniques to open surgical biopsies. Their results showed substantially lowered cost associated with percutaneous biopsy and decreased but acceptable diagnostic accuracy when compared to open biopsy [7]. However, they did not statistically quantify their results. A retrospective study performed by Adams et al. found that office-based CNB performed by fellowship-trained musculoskeletal oncologists had an 81% exact diagnostic accuracy and 97% accuracy in determining malignancy [3]. Srisawat et al. performed a retrospective chart review and found high accuracy rates of outpatient percutaneous biopsy based on malignancy (96.84%) and exact diagnosis (89.47%) [8]. Despite these adequate levels of diagnostic accuracy, a study performed by Bedi et al. found significantly increased complications when percutaneous biopsies were performed in the clinic, rather than in a specialized sarcoma center [23]. However, these results could potentially be explained by a lack of fellowship-trained musculoskeletal oncologists performing the biopsies in the community setting. In a recent review of 371 patients who underwent resection of a soft tissue mass following core needle biopsy, Strauss et al. found core needle biopsy to be able to differentiate benign versus malignant in 97.6% of cases, and to be able to identify the tumour subtype in 89.5% of benign lesions and 88% of sarcomas [19]. These cases were performed at a tertiary referral center, as opposed to the community setting in which the cases in the present study were performed.

### 4.2. Safety and Accuracy

In this study, we determined the accuracy of the outpatient percutaneous biopsy through comparison with the pathology report from the final surgical excision. We found that percutaneous biopsy was 87.0% accurate in determining the exact diagnosis of soft tissue masses and 94.2% (*p* < 0.0001) accurate in determining the status (benign versus malignant) of the masses. There were few complications related to the clinic-based percutaneous biopsy. Of the 69 patients that were included in final data analysis, 2 patients experienced pain following the initial percutaneous biopsy, 1 patient developed a hematoma at the biopsy site, and 1 patient had bleeding from the wound site requiring sutures. This resulted in a biopsy complication rate of 5.8%, which is substantially lower than the 46% reported by Bedi et al. [23] This decreased complication rate is likely attributable to the training of the treating physician, as the community biopsies performed in the referenced study were not performed by fellowship-trained musculoskeletal oncologists.

### 4.3. Clinical Utility

Using our data, we also investigated clinic-based CNB as a diagnostic tool for detecting malignancy. We determined the sensitivity, specificity, PPV, and NPV by comparing the results of the outpatient biopsy to the pathology of the final excision. The overall sensitivity was found to be 87.1% while the overall specificity was 100.0%. Overall PPV and NPV were 100.0% and 90.5%, respectively. The high level of specificity meant that we were likely to detect all benign masses as benign with our outpatient biopsy. However, our sensitivity and NPV indicate that some false negatives did exist and some malignant lesions were initially detected as benign. The specificity of our outpatient biopsy demonstrates that there were no false-positive results, and that all truly benign masses were identified as such. Our PPV shows that all masses identified as malignant by initial clinic-based CNB were identified as malignant by subsequent pathologic analysis of the excised masses. We determined these measures to be acceptable for using clinic-based biopsy as a diagnostic test, particularly when compared to results previously reported in the literature [31, 32]. In comparing treatment rendered to final histological diagnosis, all patients received appropriate operative and adjunctive treatment using this treatment algorithm, despite the small number of false-negative results.

### 4.4. Limitations

Limitations of this study centered on its retrospective nature. Retrospective chart review studies do not allow for proper randomization of subjects or control of confounding variables. Additionally, there is no potential for standardization of procedures or data collection, but this was likely mitigated through the use of a single physician's patient population. Since this chart review covered multiple years of patient visits, multiple pathologists were involved in the reporting of biopsy results. Our study design likely minimized this impact through the use of a third-party experienced pathologist in interpreting the level of agreement between the two biopsy methods. We also recognized that this study only included those patients who underwent surgical excision of their soft tissue masses following the clinic-based biopsy. This could have led to our population including a disproportionate number of patients with malignant diagnoses. However, final surgical excision pathology was needed in our study design to be used as a gold standard with which we compared our results. The best approach to this research question would involve a double-blind, randomized clinical trial in which patients were placed into one of the two biopsy techniques. However, our study design allowed for comparison of two validated biopsy techniques in the same masses, essentially using the subjects as their own control.

## 5. Conclusion

Many orthopedic surgeons believe that percutaneous biopsies of soft tissue masses should only be done in sarcoma referral centers, with concerns of decreased accuracy, potential complications, and alterations in the clinical course if biopsies are performed in the community [7, 13, 15, 23, 25]. Our results showed that diagnostic accuracy and clinical utility can be safely achieved with percutaneous core needle biopsies performed in community setting by fellowship-trained musculoskeletal oncologists. This potentially obviates the need for referral of patients with soft tissue masses to a sarcoma center, especially in cases where this may be cumbersome or costly to the patient.

## Figures and Tables

**Table 1:** 

Characteristics	Values (*n*=69)
Age (years, SD)	54.5 (18.5)

Gender (*n*, %)	
Female	31 (44.9)
Male	38 (55.1)

Location of lesion (*n*, %)	
Lower extremity	51 (73.9)
Upper extremity	18 (26.1)

Size of lesion (*n*, %)	
<5 cm	19 (27.5)
≥5 cm	46 (66.7)
Cyst/abscess	4 (5.8)

Depth (*n*, %)	
Superficial	6 (8.7)
Deep	63 (91.3)

Biopsy results (*n*, %)	
Benign	42 (62.3)
Malignant	27 (37.7)

Final pathology (*n*, %)	
Benign	38 (55.1)
Malignant	31 (44.9)
